# Evaluating Right Ventricular Function Using Longitudinal Displacement

**DOI:** 10.3390/medicina61030446

**Published:** 2025-03-03

**Authors:** Marina Leitman, Vladimir Tyomkin

**Affiliations:** 1 Department of Cardiology, Shamir Medical Center, Zerifin 70300, Israel; 2 Sackler School of Medicine, Tel Aviv University, Tel Aviv 69978, Israel

**Keywords:** right ventricular function, right ventricular displacement, manual measurement of right ventricular strain

## Abstract

*Background and Objectives:* The right ventricle has a complex, asymmetrical shape, making accurate imaging and functional assessment by echocardiography challenging. Various methods have been proposed for evaluating right ventricular function, each one with its limitations. This study introduces a new method for assessing global and regional right ventricular function using longitudinal displacement. *Materials and Methods:* We studied 21 healthy young individuals who underwent echocardiographic examinations at our hospital for screening purposes. Speckle-tracking echocardiography was used to analyze their echocardiographic images and measure the longitudinal displacement of the right ventricle. *Results:* Our findings show that longitudinal displacement is highest in the basal segments and lowest in the apical segments of the right ventricle, demonstrating a “reversed basal-to-apical gradient”. Longitudinal strain, on the other hand, was found to be highest at the apex and lowest at the base. We observed a strong correlation between longitudinal displacement and tricuspid annulus plane excursion (TAPSE), with an agreement of 89.47%. Longitudinal displacement over the right ventricle free wall was significantly higher than that over the septum. There was a good agreement between the manual and automatic measurements of right ventricular strain. *Conclusions:* Longitudinal displacement of the right ventricle can be reliably measured using speckle-tracking—echocardiography. This original measurement provides a “true” assessment of displacement at each right ventricular segment without postprocessing. Unlike TAPSE, which measures tricuspid annular motion, longitudinal segmental displacement offers comprehensive data on all segments at each level and can serve as an additional tool for assessing right ventricular function. The manual assessment of right ventricular strain provides a practical option in appropriate clinical settings.

## 1. Introduction

The right ventricle (RV) is a vital component of the cardiovascular system, responsible for pumping deoxygenated blood into the lungs for oxygenation. Proper assessment of RV function is essential, as RV dysfunction can contribute to significant clinical conditions, including heart failure, pulmonary hypertension, and arrhythmias [1]. Due to its unique, crescent-shaped, and asymmetrical anatomy, accurate imaging and assessing RV function can be challenging using conventional echocardiographic techniques [2,3]. Unlike the left ventricle, which consists of three distinct myocardial layers, the right ventricle does not have a well-defined middle layer. The superficial layer, making up about 25% of the wall thickness, is primarily composed of circumferential fibers that run parallel to the atrioventricular groove and extend between the ventricles. The endocardial layer of the right ventricle consists of longitudinally oriented myocytes that extend from the apex toward the papillary muscles, tricuspid annulus, and right ventricular outflow tract, ensuring coordinated contraction and maintaining continuity with the septal myocardium. This structural integration highlights the close anatomical relationship between the right and left ventricles. Both ventricles share common circumferential fibers and occupy the same pericardial cavity, which facilitates their mechanical interaction and underlies their physiological interdependence. This interplay is crucial for maintaining balanced ventricular function and optimizing cardiac output [4]. Right ventricular myocytes are smaller in size compared to those in the left ventricle, and the right ventricular myocardium has a higher collagen content, contributing to its unique structural and mechanical properties [5]. The right ventricle has a 10–15% larger volume than the left ventricle but features a thinner free wall, measuring approximately 3–5 mm. This structural difference reflects its role in handling a lower-pressure pulmonary circulation [6,7]. Right ventricular coronary flow is lower than that of the left ventricle and takes place during both systole and diastole. Because of its thinner wall and increased dependence on coronary perfusion pressure, the right ventricle is more prone to dysfunction in cases of systemic hypotension [8]. Traditional metrics for assessing right ventricular function, such as tricuspid annular plane systolic excursion (TAPSE), fractional area change (FAC), and three-dimensional ejection fraction (3D EF), are widely utilized in clinical practice. TAPSE is a widely recognized measure of RV longitudinal function that reflects the motion of the tricuspid annulus during systole. However, TAPSE alone is limited because it does not provide a comprehensive view of RV function across the entire chamber [9]. FAC evaluates the change in RV area during the cardiac cycle but may not accurately capture the RV’s regional function or reflect subtle changes in contractility [5]. While 3D EF provides an improved assessment by quantifying RV volume and ejection fraction, its accessibility remains limited due to the complexity of acquisition and the challenges in controlling automatic measurements [10,11].

Strain imaging, specifically using speckle-tracking echocardiography, has emerged as a valuable tool for assessing myocardial function, including RV strain. Strain measures the deformation of myocardial fibers during the cardiac cycle, offering insights into both the global and regional RV function [12]. Longitudinal strain, in particular, is sensitive to subtle changes in RV function and provides a more detailed understanding of RV contractility compared to traditional metrics [13,14]. Regional differences in strain values, such as those observed in the basal and apical segments, can help identify localized dysfunction not captured by global assessments. However, in contrast to the left ventricle, regional strain analysis of the right ventricle has not been extensively studied. Limited data exist on the basal-to-apical gradient of RV strain. One study identified this gradient in children, suggesting regional heterogeneity in RV strain [15]. However, to date, segmental strain differences in the RV have not been systematically evaluated in adults. Recognizing the complexities of RV assessment, this study introduces longitudinal displacement as a novel metric for evaluating global and regional RV function. This approach provides segmental data without postprocessing influence, offering a direct measurement of displacement at each RV segment. Considering the difficulties in precisely evaluating right ventricular function, longitudinal displacement, which serves as a precursor of strain, holds promise as an effective technique for assessing right ventricular function.

We aim to compare this innovative method to TAPSE and explore its potential as an additional tool for comprehensive RV function assessment.

## 2. Materials and Methods

A total of 21 young healthy male individuals underwent echocardiography examination in our echolab for screening purposes. Echocardiographic examination of these subjects were retrieved and analyzed offline using speckle-tracking imaging. Global and regional longitudinal strain and global and regional longitudinal displacement was calculated for each subject.

### 2.1. Echocardiographic Assessment

All echocardiographic examinations were conducted using the Vivid E95 ultrasound system (General Electric, Horten, Norway) equipped with a transducer operating at a frequency range of 1.7–4 MHz. The imaging frame rate was maintained at a minimum of 50 frames per second. Comprehensive transthoracic echocardiography was performed following the most recent guidelines for chamber quantification [16]. This included linear, volumetric, and Doppler assessments. Standard imaging views were obtained, including parasternal long-axis and short-axis views at basal, mid-ventricular, and apical levels, as well as apical 4-chamber, 2-chamber, and 3-chamber views.

The biplane left atrial volume index (*LAVi*) was determined using the following equation:

$$LAVi=(\frac{8}{3}\pi \times \frac{\left(A1\right)\times \left(A2\right)}{L})/BSA$$

where *A*1 and *A*2 represent the left atrial areas measured in the apical 4-chamber and 2-chamber views, respectively, *L* refers to the left atrial length, and *BSA* stands for body surface area.

The left ventricular mass index (*LVMi*) was determined using the following equation:

$$LVMi=(0.8\times 1.4\times [{\left(IVS+PW+LVID\right)}^{3}-{LVID}^{3}]+0.6\;g)/BSA$$

where *IVS* denotes the thickness of the interventricular septum at end-diastole, *PW* refers to the thickness of the posterior wall at end-diastole, and *LVID* indicates the internal dimension of the left ventricle at end-diastole. *BSA* represents body surface area.

Diastolic function was evaluated in accordance with current recommendations [17], incorporating measurements of E wave and A wave amplitudes, the E/A ratio, E wave deceleration time, and tissue Doppler parameters such as septal E′ velocity (E′s) and lateral E′ velocity (E′l).

All echocardiographic examinations were then transferred to the EchoPAC workstation (Version 204) for further offline speckle-tracking imaging analysis. Speckle-tracking imaging analysis was performed according to the original recommendations using the right ventricle-focused apical four-chamber view [18]. The region of interest included both the right ventricle free wall and septum.

Doppler tracings of the tricuspid and pulmonary valves were utilized to define the timing of end-diastole and end-systole, respectively [13]. Pulmonary valve closure, confirmed through pulmonic Doppler flow obtained from the parasternal short-axis view, was used as the reference point for end-systole.

Offline speckle-tracking analysis was conducted to assess global and regional longitudinal displacement, as well as global and regional longitudinal strain, of the right ventricle.

### 2.2. Specification Regarding Longitudinal Deformation Parameters of the Right Ventricle: Strain and Displacement, Figure 1

Strain refers to relative deformation, determined by the ratio of the change in length to the original length, and is reported as a percentage [19].

**Figure 1:**
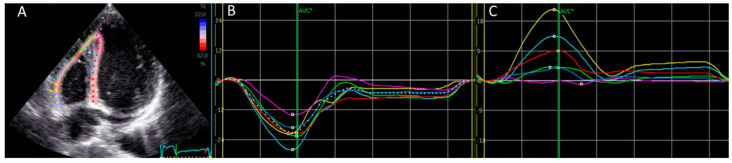
Calculation of global and regional longitudinal right ventricular displacement. (**A**) Region of interest. Region of interest includes right ventricular free wall a and interventricular septum. (**B**) Right ventricular strain. Right ventricular longitudinal strain from the six segments has negative direction. (**C**) Right ventricular displacement. In contrary to strain, right ventricular displacement from six cardiac segments has position direction.

Strain is determined using the following formula:

$$Strain=\frac{L\left(t\right)-L\left(0\right)}{L\left(0\right)}$$

where *L*(0) represents the initial length and *L*(*t*) denotes the length at a given point in time. Longitudinal strain is generally negative, as the right ventricle undergoes shortening during systole (Figure 1A,B).

Longitudinal displacement refers to the absolute change in length, measured in millimeters (mm), and is calculated using the following formula:

$$Displacement=L\left(t\right)-L\left(0\right),$$

where *L*(0) is the initial length and *L*(*t*) is the length at a specific time.

Typically, longitudinal displacement in the right ventricle is positive, indicating movement in the direction of systolic shortening (Figure 1C).

### 2.3. Manual Calculation of Global Right Ventricular Strain

**Figure 2:**
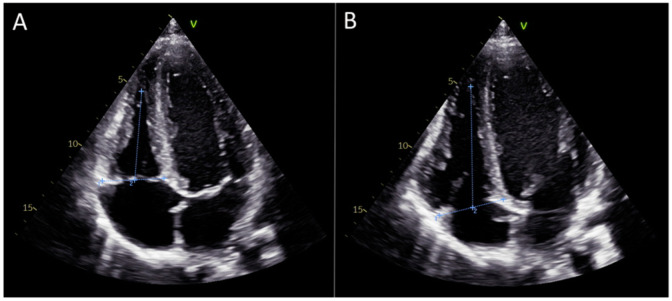
Measurements necessary for the manual calculation of right ventricular strain. (**A**) The length of the right ventricle was measured from the tricuspid annulus plane to the apex of the right ventricle at the end of systole. (**B**) The length of the right ventricle was measured from the tricuspid annulus plane to the apex of the right ventricle at the end of diastole.

For this purpose, the length of the right ventricle was measured from the tricuspid annulus plane to the apex of the right ventricle at the end of systole and diastole, corresponding to the maximal shortening and maximal lengthening, respectively. These measurements were taken from the same 4-chamber view used for strain calculation (Figure 2A,B).

The manual right ventricular strain was then calculated using the following formula:

$$Strain(m)=\left(\frac{Ls-Ld}{2}\right)\times 100$$

where (*m*) denotes manual measurement, (*Ls*) is the length of the right ventricle at the end of systole, and (*Ld*) is the length of the right ventricle at the end of diastole.

### 2.4. Statistical Methods

Descriptive statistics were used to summarize the characteristics of each parameter. Continuous data are expressed as means ± standard deviations. The normality of data distributions was assessed using the Kolmogorov–Smirnov test. A two-tailed dependent *t*-test was applied to compare continuous variables. Categorical data are presented as numbers and percentages. Univariate analysis was performed using the Chi-Square test or Fisher’s exact test, as appropriate, to identify significant variables (*p* < 0.05). All statistical analyses were conducted using IBM SPSS Statistics for Windows, Version 28.0 (Armonk, NY, USA: IBM Corp).

### 2.5. Ethical Approval

This retrospective study was based on patient records, and formal approval from the Ethics Committee (Helsinki, Finland) at Shamir (Assaf Harofeh) Medical Center was not required. Informed consent was also not necessary. Additionally, all images included in the article have been anonymized to ensure patient confidentiality. All procedures were conducted in accordance with applicable guidelines and regulations.

## 3. Results

The demographic and echocardiography parameters are presented in Table 1. The mean age of patients was 24.8 ± 5.0 and echocardiography parameters within normal limits.

**Figure 3:**
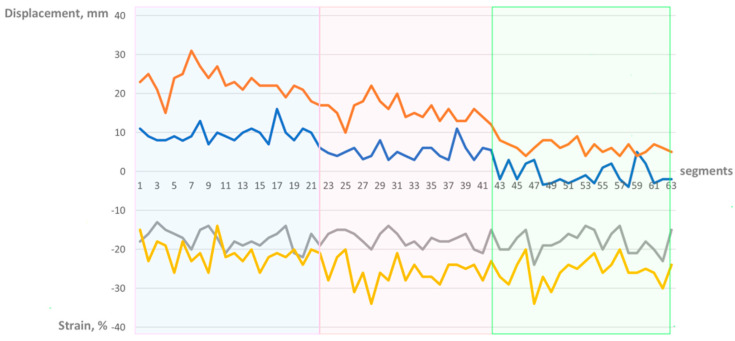
Right ventricular free wall versus septum, displacement and strain. Brown line—free wall displacement, Blue line—septal displacement, Yellow line—free wall strain, Grey line—septal strain. Blue background—basal segments, Pink background—midventricular segments, Green background—apical segments. Free wall longitudinal displacement is higher than the septal. Free wall strain is higher than the septal.

Speckle-tracking imaging analysis of this group showed that displacement was highest in the basal segments of the right ventricle, free wall and septum, and lowest in the apex (Table 2, Figure 3). Free wall longitudinal displacement is higher than the septal. Right ventricular strain in contrary was lowest at the basal segments and highest in mid and apical segments (Table 2, Figure 3). Free wall right ventricular strain is higher than the septal.

Right ventricular displacement is highest at the basal segments and lowest at the apex in both the free wall and interventricular septum.

In contrast, right ventricular strain is the lowest at the basal segments. No significant difference in strain measurements is observed between the mid and apical segments.

Free wall longitudinal displacement was higher than the septal 14.8 ± 7.3 mm vs. 4.6 ± 4.8 mm, *p* < 0.000001, as well as free wall longitudinal strain was higher than the septal −24.2% ± 3.9 vs. −17.6% ± 2.4, *p* < 0.000001.

**Figure 4:**
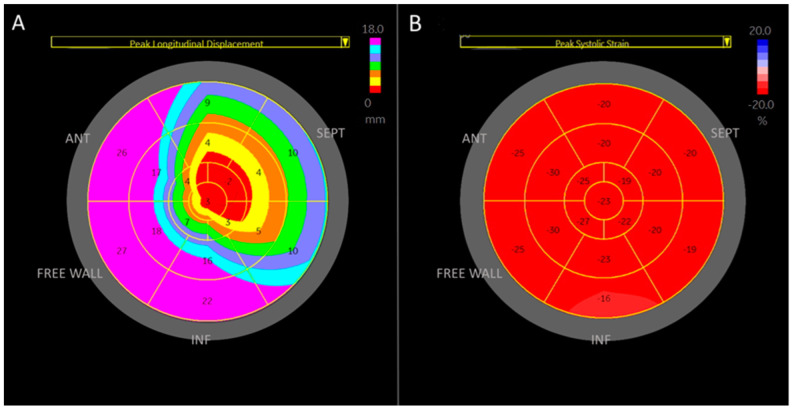
Visual plots of longitudinal displacement and strain for the right ventricle, extrapolated from the apical four-chamber view. (**A**) Visual plot of longitudinal displacement. Longitudinal displacement is highest in the basal segments of the right ventricle and lowest in the apical segments. (**B**) Visual plot of longitudinal strain. Longitudinal strain is lowest in the basal segments of the right ventricle. The schematic nomenclature of the right ventricle regions was used: Ant—anterior region, Inf—inferior region, Sept—septal region, and free wall.

A visual plot was extrapolated from the apical focused view to the whole right ventricle for longitudinal displacement and strain (Figure 4). The highest displacement was seen in the basal segments of the right ventricle, the lowest displacement was found at the apex. Septal displacement is lower than in the free wall. Strain was the lowest in the basal segments of the right ventricle.

**Figure 5:**
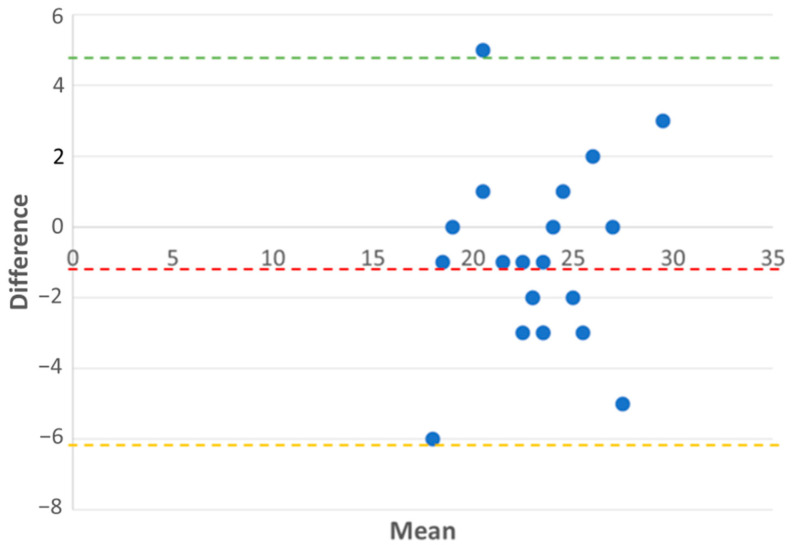
Bland-Altman analysis of agreement between longitudinal displacement of the basal right ventricular segment and TAPSE. x-axis: Mean of longitudinal displacement of the basal right ventricular segment and TAPSE. y-axis: Difference between longitudinal displacement of the basal right ventricular segment and TAPSE. Upper limit of agreement: 4.96, Lower limit of agreement: −5.96. The agreement percentage is 89.47%, indicating a high level of agreement between the two methods. Dashed green and yellow lines represent the limits of agreement, with the central red line denoting the mean difference.

Based on the determination, the longitudinal displacement of the basal free wall segment is TAPSE—tricuspid annular systolic excursion. We conducted an analysis and comparison of TAPSE, measured by conventional way using M-mode and longitudinal displacement of the whole basal segment using speckle-tracking analysis. Bland–Altman analysis of agreement between longitudinal displacement of the basal right ventricular segment and TAPSE was 89.47%, showing a high level of agreement between two methods (Figure 5). On average, TAPSE overestimates right ventricular function by 1 mm compared to the displacement of the basal right ventricular free wall segment, which represents a small bias (Table 3).

**Figure 6:**
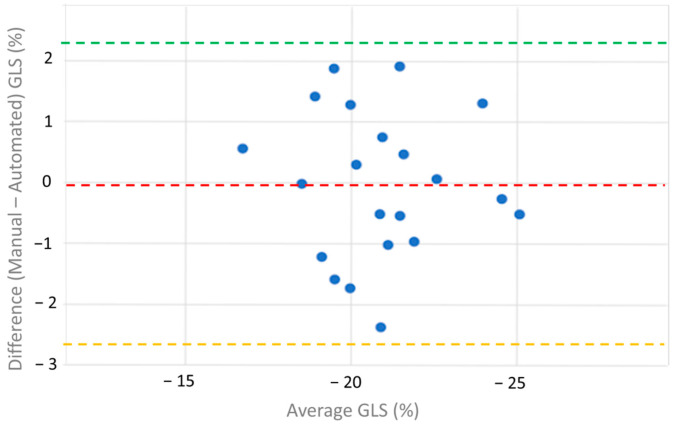
Bland-Altman plot comparing manually measured right ventricular strain and automated (speckle-tracking-imaging-based) right ventricular strain. The x-axis represents the average right ventricular strain (%) between two methods, while the y-axis shows the difference (manual-automated) right ventricular strain (%). The red dashed line indicates the mean difference (−0.04%), closed to zero. The green and yellow dashed lines represent the upper (2.27%) and lower (−2.68%) limits of agreement, respectively, showing good agreement between the methods.

The Bland–Altman plot demonstrates strong agreement between manual and automated speckle-tracking imaging-based measurements of right ventricular strain, with minimal bias and narrow limits of agreement (Figure 6). The calculated bias is −0.04 (Table 4).

## 4. Discussion

In this work, we propose an additional method for evaluating right ventricular (RV) function: longitudinal displacement. Our findings reveal inhomogeneity in longitudinal displacement within the normally functioning right ventricle. Specifically, the highest longitudinal displacement was observed in the basal segments, while the lowest displacement occurred at the apex. This pattern demonstrates a reversed basal-to-apical gradient for longitudinal displacement.

In contrast, right ventricular strain exhibited the opposite gradient, with the highest strain values at the apex and the lowest at the base. While left ventricular strain is typically highest at the apex and lowest at the base [20], regional right ventricular (RV) deformation parameters have been largely underexplored. The right ventricle predominantly consists of longitudinal fibers, and longitudinal shortening contributes approximately 75% of RV contraction. Additionally, the left ventricle plays a supportive role, contributing 20–40% to RV ejection through the interventricular septum [21].

The literature on regional RV strain highlights significant variability, with reported values differing by 30–40%, leading to its limited recommendation for clinical use [13,21]. Notably, only two studies have specifically investigated segmental RV strain. One study in adults using vector velocity imaging identified a basal-to-apical gradient [22], while a study in children employing 2D speckle-tracking imaging found a similar gradient [15]. However, another study reported uniform regional strain across different RV levels [23]. Additional investigations have produced inconsistent controversial findings, with some studies showing basal strain as the highest and apical strain as the lowest [24,25,26].

These studies were conducted using earlier generations of echocardiography systems. Strain is not a direct measurement but rather a derived parameter calculated through formulas that manipulate displacement data. When the region of interest is not adequately defined or segment tracking is inaccurate, even minor errors in displacement measurements can result in significant inaccuracies in strain assessment. This issue is particularly relevant for the right ventricle, as obtaining a properly focused view suitable for accurate strain analysis is often challenging.

For this reason, displacement offers a more fundamental parameter, directly measured by software, similar to TAPSE. In our study, longitudinal displacement demonstrated a reversed basal-to-apical gradient. Calculated strain was highest at the apex and lowest at the base, consistent with the findings of two previously mentioned studies [15,22], one conducted on children and the other on adults using vector velocity imaging.

The strong agreement between basal segment longitudinal displacement and TAPSE supports the reliability of longitudinal displacement as a parameter. A meticulous evaluation of segmental right ventricular function provides deeper insights into the physiology of right ventricular contraction. The model of right ventricular displacement and strain that we developed by extrapolating our results aids in visualizing and understanding the correct functional mechanics of the right ventricle.

A strong agreement was observed between manual and speckle-tracking-imaging-based measurements of right ventricular strain. The small mean difference and narrow limits of agreement indicate that the two methods are interchangeable, particularly in scenarios where automated software is unavailable or unsuitable.

Evaluating right ventricular function through longitudinal displacement can provide important insights into different pathological conditions. The right ventricle may undergo pressure and volume overload and can also be influenced by primary myocardial diseases. Pressure overload frequently arises from chronic pulmonary hypertension and acute conditions like pulmonary embolism. Accurate diagnosis is essential in cases of pulmonary embolism, as echocardiography is typically the first diagnostic tool for patients presenting with dyspnea. The longitudinal displacement technique can assess slight systolic flattening of the interventricular septum caused by acute pressure overload, potentially enabling early diagnosis before significant right ventricular dysfunction occurs.

In the chronic phase of pressure overload, the right ventricle initially adapts through concentric remodeling, where the muscle wall thickens in response to increased stress, allowing it to maintain normal function. This adaptive process helps preserve cardiac output and prevent overt dysfunction. However, over time, as the pressure overload persists, the heart undergoes further changes. In more advanced stages, the right ventricle may develop eccentric hypertrophy, where both the muscle wall thickens and the chamber dilates. This process represents maladaptive remodeling, which compromises the efficiency of right ventricular function and may eventually lead to right ventricular failure. The shift from adaptive concentric remodeling to maladaptive eccentric hypertrophy is a key transition point, marking the progression from compensated to decompensated right ventricular dysfunction [3]. Prolonged volume overload causes alterations in the configuration and motion of the interventricular septum, which ultimately leads to systolic dysfunction. This dysfunction can be identified earlier through displacement techniques, helping to detect changes before they become more severe and enabling timely intervention for congenital cardiac anomalies.

A “cardiomyopathic right ventricle” [3] encompasses a variety of diseases that primarily affect the right ventricular myocardium. Right ventricular systolic function can be compromised in cases of inferior myocardial infarction, which involves the right ventricle in approximately 50% of cases [27]. Right ventricular involvement is also observed in acute myocarditis [28] and is a characteristic feature of cardiac amyloidosis [29], where amyloid deposition in the right ventricle forms the anatomical foundation for right ventricular dysfunction [30]. In hypertrophic cardiomyopathy, the right ventricle may be affected due to the involvement of the septum and as a result of pulmonary hypertension that develops from diastolic dysfunction. In sarcoidosis, right ventricular damage may arise as a result of pulmonary hypertension, which can develop due to the restrictive effects of granulomas on lung tissue and vasculature. This pulmonary hypertension can, in turn, place increased strain on the right ventricle, leading to its dysfunction. Additionally, sarcoidosis can directly affect the right ventricular myocardium through the formation of granulomas, which may impair myocardial contractility and contribute to right ventricular failure. Therefore, right ventricular damage in sarcoidosis can occur from both secondary pulmonary hypertension and the primary involvement of the myocardial tissue by the disease, often complicating the overall clinical picture [3]. Longitudinal displacement can be a valuable tool for diagnosing early right ventricular dysfunction in these conditions. Arrhythmogenic right ventricular cardiomyopathy (ARVC) may remain in a subclinical or concealed phase for an extended period, only becoming apparent in later stages when electrical abnormalities emerge. This is followed by structural changes, including overt right ventricular dysfunction and dilation [31]. Early diagnosis of ARVC during the asymptomatic stage is essential and may be achievable through the use of longitudinal displacement, which can help detect subtle functional changes before structural abnormalities become evident.

Differentiating stress-induced ARVC from right ventricular remodeling in athletes can be particularly challenging [32,33]. This ARVC-like phenotype, often referred to as Phidippides cardiomyopathy in endurance athletes [34], may develop as a result of chronic pressure and volume overload due to intense exercise. Exercise plays a significant role as an environmental factor in the pathogenesis of ARVC, and prolonged athletic activity may contribute to the acceleration of disease progression. Therefore, accurate diagnosis and the ability to differentiate between an athlete’s heart and ARVC are crucial, and longitudinal displacement could be a helpful tool in making this distinction.

Further research utilizing longitudinal displacement is recommended to enhance the diagnosis of right ventricular dysfunction in a range of cardiac and multisystemic pathological conditions.

### Limitations

The small sample size of 21 healthy subjects restricts the generalizability of our findings to the healthy population. Further studies with larger and more diverse cohorts are needed to validate these results and explore their applicability in clinical settings.

## 5. Conclusions

The longitudinal displacement of the right ventricle can be reliably assessed using speckle-tracking imaging. This novel measurement provides an accurate evaluation of displacement at each right ventricular segment without the need for postprocessing. Unlike TAPSE, which measures tricuspid annular motion, longitudinal segmental displacement offers detailed data on all segments at each level, making it a valuable additional tool for assessing right ventricular function.

Manual assessment of right ventricular strain is a viable alternative tailored to specific clinical scenarios.

## Figures and Tables

**Table 1:** Demographic and echocardiographic characteristics of the study population (normal subjects).

Number	21
Age, years	24.8 ± 5.0
Height, cm	182.7 ± 6.2
Weight, kg	76.5 ± 6.1
BSA m^2^	1.98 ± 0.1
LAVi, mL/m^2^	35.3 ± 8.2
LVEDD, cm	5.1 ± 0.4
LVESD, cm	3.2 ± 0.4
IVS, cm	0.9 ± 0.1
PW, cm	0.9 ± 0.1
LVMi g/m^2^	85.8 ± 17.1
EF, %	58.8 ± 3.1
E/A	1.9 ± 0.5
E dec, msec	173.4 ± 48.2
E/E’ ratio	4.7 ± 0.6

BSA—body surface area; LAVi—left atrial volume index; LVEDD—left ventricular end-diastolic diameter; LVESD—left ventricular end-systolic diameter; IVS—interventricular septum thickness; PW—posterior wall thickness; LVMi—left ventricular mass index; EF—ejection fraction; E/A—early (E) to late (A) diastolic transmitral flow ratio; E dec—E-wave deceleration time; E/E’—ratio of early mitral inflow velocity to early diastolic mitral annular velocity.

**Table 2:** Longitudinal right ventricular displacement and strain across six segments.

Variable	Displacement, mm	*p*-Value	*p*-Value	
Free wall basal	22.8 ± 3.3	NA	21.2 ± 3.2	NA
Free wall mid	15.6 ± 2.7	<10(−8)	25.7 ± 3.4	<10(−4)
Free wall apical	6.1 ± 1.5	<10(−16)	25.6 ± 3.4	0.9
Septal basal	9.6 ± 2.0	NA	18.2 ± 2.8	NA
Septal mid	5.1 ± 1.9	<10(−9)	17.3 ± 1.9	0.27
Septal apical	(−)0.8 ± 2.5	<10(−9)	17.2 ± 2.4	0.89
Global value	9.9 ± 1.3	NA	20.9 ± 2	NA

NA—not applicable.

**Table 3:** Comparison between the displacement of the basal right ventricular segment and TAPSE measurements.

Measurement	Displacement of the Basal RV Segment	TAPSE
Mean ± SD, mm	22.76 ± 3.29	23.76 ± 3.02
Outliers	2 (9.5%)	1 (4.8%)
Inliers	19 (90.5%)	20 (95.2%)
Bias	1 (TAPSE overestimates by 1 mm)	NA

Outliers—Measurements outside the limits of agreement (mean ± 2SD). Inliers—Measurements within the limits of agreement (mean ± 2SD). Bias—1 mm, indicating that on average, TAPSE overestimates right ventricular function by 1 mm compared to the displacement method, a small bias. NA—not applicable.

**Table 4:** Comparison of manual and automatic right ventricular strain measurement.

Measurement	Manual RV Strain	Automatic RV Strain
Mean ± SD, %	−20.90 ± 2.08	−20.08 ± 2.03
Outliers	2 (9.5%)	0 (0%)
Inliers	19 (90.5%)	21(100%)
Bias	−0.04	NA

Outliers: Measurements outside the limits of agreement (mean ± 2SD). Inliers: Measurements within the limits of agreement (mean ± 2SD). Bias: The mean difference between the manual and automatic methods (−0.04), indicating minimal bias.

## Data Availability

The data supporting the findings of this study are available from the corresponding author upon reasonable request.
